# Daily Lifestyle and Inflammatory Skin Diseases

**DOI:** 10.3390/ijms22105204

**Published:** 2021-05-14

**Authors:** Yu Sawada, Natsuko Saito-Sasaki, Emi Mashima, Motonobu Nakamura

**Affiliations:** Department of Dermatology, University of Occupational and Environmental Health, 1-1, Iseigaoka, Yahatanishi-Ku, Kitakyushu 807-8555, Japan; natsuko-saito@med.uoeh-u.ac.jp (N.S.-S.); e-mashima@med.uoeh-u.ac.jp (E.M.); motonaka@med.uoeh-u.ac.jp (M.N.)

**Keywords:** daily lifestyle, psoriasis, atopic dermatitis, contact dermatitis, skin inflammation

## Abstract

Throughout life, it is necessary to adapt to the Earth’s environment in order to survive. A typical example of this is that the daily Earth cycle is different from the circadian rhythm in human beings; however, the ability to adapt to the Earth cycle has contributed to the development of human evolution. In addition, humans can consume and digest Earth-derived foods and use luxury materials for nutrition and enrichment of their lives, as an adaptation to the Earth’s environment. Recent studies have shown that daily lifestyles are closely related to human health; however, less attention has been paid to the fact that obesity due to excessive energy intake, smoking, and alcohol consumption contributes to the development of inflammatory skin diseases. Gluten or wheat protein, smoking and alcohol, sleep disturbance, and obesity drive the helper T (Th)1/Th2/Th17 immune response, whereas dietary fiber and omega-3 fatty acids negatively regulate inflammatory cytokine production. In this review, we have focused on daily lifestyles and the mechanisms involved in the pathogenesis of inflammatory skin diseases.

## 1. Introduction

Human beings need to carry out fundamental actions such as food consumption and sleep, i.e., a daily lifestyle, which affects physical, pathological, and psychological health conditions [1,2]. Hypertension and diabetes are well recognized as daily lifestyle-related diseases [3], and recent studies have shown that inflammatory diseases are also closely associated with daily lifestyle factors [4].

**Figure 1:**
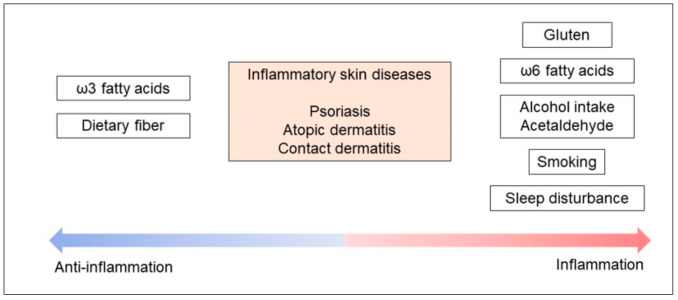
Inflammatory skin diseases are associated with daily lifestyle factors. This schema showed representative daily lifestyle factors related to the activation/regulation of inflammatory skin diseases.

The skin is the outermost organ in the human body and is exposed to various environmental factors [5,6,7]. These factors influence the homeostasis of physiological skin function and sometimes induce an inflammatory response in the skin for appropriate adaptation to external environmental stimuli [8]. For instance, psoriasis is a representative inflammatory disease in the dermatology field, and daily lifestyle is well understood to exacerbate or regulate cutaneous inflammation [9,10]. Internal physiological factors also influence the degree of skin inflammation [11]. Patients themselves have recognized the relationship between skin inflammation and internal physiological factors; they also recognize how difficult it is to change their daily lifestyles to control inflammatory skin diseases [12]. Therefore, knowledge of the daily lifestyle associated with inflammatory skin disease is an important factor for clinicians and researchers and is inseparable from the actual clinical practice of managing such diseases. However, the intrinsic and extrinsic environmental influences of daily lifestyle on skin inflammation have not been well summarized in prior review articles. In this review, we first introduce representative skin disease pathogenesis and then summarize the daily lifestyle influence on inflammatory skin diseases (Figure 1). We believe that the instructions concerning daily lifestyle and inflammatory skin disease will be helpful for clinicians to treat patients with intractable skin conditions.

## 2. Pathogenesis of Inflammatory Skin Diseases

### 2.1. Psoriasis

Psoriasis is a representative chronic inflammatory skin disease characterized by epidermal proliferation and infiltration of inflammatory immune cells. Tumor necrosis factor (TNF)-mediated interleukin (IL)-23/IL-17 involvement has been identified in its pathogenesis, and the importance of these pathways has been proven using various targeted biological drugs [13,14]. A mouse model, the imiquimod-induced psoriasis model, mimics human psoriasis skin lesions, and has helped develop an understanding of the pathogenesis of psoriasis based on IL-23/IL-17-mediated inflammatory skin lesions [15]. The IL-17-mediated pathway enhances inflammatory reactions in the epidermis, and keratinocytes produce various inflammatory cytokines, chemokines, and antimicrobial peptides [16].

Logically, based on the pathogenesis of psoriasis, these cytokine-targeted drugs should completely suppress skin inflammation; however, not all patients necessarily exhibit complete response to biologics, and psoriasis patients experience recurrence of skin eruption [17]. In that case, the excessive burden of working style or lifestyle, such as shift working, might contribute to the development of psoriasis skin inflammation. In addition, IL-17 contributes to the development of cutaneous defense against microorganisms. In fact, the treatment with anti-IL-17 antibody drugs shows a higher frequency of mucocutaneous candidiasis [18]. Although biologics treatment is useful for the treatment of psoriasis, these findings suggest the importance of daily lifestyle guidance for patients with psoriasis, in addition to the current treatment.

### 2.2. Atopic Dermatitis

Skin barrier disruption causes the entry of external antigens into the skin [19]. One of the reasons for skin barrier disruption is related to the filaggrin gene mutation [20,21]. Within the stratum corneum, filaggrin is essential for skin barrier function by integrating this monomer into the lipid envelope [22]. In addition, filaggrin is processed additionally in the upper stratum corneum to release free amino acids, which contribute to retaining the moisturization of the skin [22]. Skin barrier disruption activates keratinocytes and Langerhans cells to produce pro-inflammatory cytokines, such as thymic stromal lymphopoietin (TSLP) and IL-33, and subsequently enhances helper T (Th) 2 immune reactions mediated by IL-4, IL-12, and IL-13 [23]. Furthermore, IL-31 accelerates itch in atopic dermatitis and decreased the quality of life [24,25]. Therefore, these cytokine-targeted treatments or the recovery of skin barrier function have been developed for the favorable clinical outcome of atopic dermatitis treatment [26,27]. In addition to the external trigger that causes atopic skin inflammation, intrinsic factors are involved in the pathogenesis of intrinsic atopic dermatitis [11]. The serum nickel concentration is constitutionally higher in patients with intrinsic atopic dermatitis than in those with extrinsic atopic dermatitis and healthy subjects [28]. In contrast to keratinocytes, fibroblasts express a high degree of Toll-like receptor (TLR) 4, which produces IL-8 in response to nickel or cobalt [29].

### 2.3. Contact Dermatitis

The pathogenesis of contact dermatitis is divided into two phases: the sensitization phase and the elicitation phase [30,31]. In the sensitization phase, external antigens exposed to the epidermis promote the expression of various inflammatory cytokines and chemokines, such as IL-1β and TNF-α, which enhance cutaneous dendritic cell activation, and upregulate the intake of antigens to prepare for maturation and migrate into the draining lymph node. In the draining lymph node, activated dendritic cells present antigens to naïve T cells to initiate the differentiation and proliferation of effector T cells according to the appropriate direction of the antigen-specific immune response. In the elicitation phase, re-exposure to antigens drives keratinocyte-derived cytokine production and activates antigen-specific T cell infiltration and activation in the antigen-exposed skin site [30]. These antigen-specific T cells produce inflammatory cytokines, such as interferon (IFN)-γ, and subsequently enhance the local inflammatory responses. In both sensitization and elicitation phases, other immune cells, such as Tregs and mast cells, are also involved in the pathogenesis of contact dermatitis [32].

## 3. Daily Lifestyle and Inflammatory Skin Diseases

### 3.1. Gluten

Gluten is a protein derived from seeds found in cereal grains and has been demonstrated to trigger celiac disease, enhancing allergic reactions to specific epitopes [33]. Gluten has also been shown to exacerbate inflammatory skin diseases in recent studies.

The involvement of gluten in the pathogenesis of psoriasis was first identified in patients with celiac disease, which is an autoimmune intestinal disease triggered by an immune response against gluten. Unexpectedly, psoriasis symptoms were also alleviated by a gluten-free diet in patients with non-celiac disease [9], suggesting that gluten might contribute to the development of psoriasis. In fact, a gluten-free diet decreases the level of growth factors and inflammatory cell infiltration in the skin [34]. Although the detailed mechanism of skin inflammation enhancement in psoriasis by gluten remains unclear, the presence of Th17 cells was identified in the duodenal mucosa of patients with celiac disease. IL-17 was upregulated in the mucosal lesions in celiac disease [35], suggesting that gluten might enhance the IL-17-dominant immune reaction in the skin.

The prevalence of atopic dermatitis is high in adults [36] and children [37] with celiac disease. In addition, gluten intake increases the risk of atopic dermatitis [38]. Gluten administration exacerbated atopic dermatitis-like skin inflammation in a mouse experiment [39]. Gluten promotes keratinocyte-derived TSLP, which enhances the Th2 immune response in the skin [40]. Therefore, these findings reasonably support the caSuse of allergic skin inflammation in patients with atopic dermatitis.

A limited number of studies have focused on the relationship between gluten and contact dermatitis. Interestingly, research shows that non-celiac wheat sensitivity is closely related to contact hypersensitivity [41]. A prospective analysis evaluated the prevalence of contact hypersensitivity to nickel, which is one of the representative agents that cause contact dermatitis, and revealed that 10% of patients with non-celiac wheat sensitivity exhibit contact dermatitis and nickel allergy with a significantly higher risk compared with control subjects.

### 3.2. Dietary Fiber

Dietary fiber is closely associated with anti-inflammatory activity in the human body. After the intake of dietary fiber, the gut microbiome improves and regulates inflammatory reactions in various inflammatory diseases [42,43]. In addition, short-chain fatty acids are produced from a dependent product of dietary fiber by specific microbes under fermentation conditions in the colon. Short-chain fatty acids can change the chromatin structure using histone modification, which can regulate gene expression without changing the deoxyribonucleic acid (DNA) sequence itself, and create open chromatin sites in specific genes, leading to enhanced gene expression [8].

Low intake of dietary fiber is associated with the development of psoriasis [10]. In addition, a high-fiber diet reduces the severity of skin scores (PASI score) in patients with psoriasis [10]. Dietary seaweed fiber intake relieves psoriatic inflammation by changing the community composition of major intestinal opportunistic microbiota [44]. Therefore, dietary fiber intake guidance is important for patients with psoriasis. As to the mechanism, short-chain fatty acids metabolized from dietary fiber increase the level of regulatory T cells, resulting in the enhancement of anti-inflammatory action [45]. Topical application of butyrate inhibits imiquimod-induced psoriasis-like skin inflammation [46].

The content of short-chain fatty acid-producing bacterial species in the gut is low in patients with atopic dermatitis [47]. Butyrate administration suppressed atopic dermatitis-like skin inflammation in mouse skin; this elucidates the importance of short-chain fatty acids in the pathogenesis of atopic dermatitis [48].

Short-chain fatty acids derived from bacteria regulated contact hypersensitivity reactions in mouse experiments during the elicitation phase [49]. Butyrate upregulates Treg function to suppress cutaneous inflammation via histone modification in an acetylation-dependent manner. In addition, the dietary fiber component, fructo-oligosaccharide, metabolized by intestinal *Bifidobacterium pseudolongum,* also suppresses contact hypersensitivity reactions in mice [50].

### 3.3. Omega (ω)-3 and Omega (ω)-6 Fatty Acids

Fatty acids are major structural components to all cells, and it also serves as signaling messengers within a cell or among different cells [51]. Since fatty acids are widely involved in the pathogenesis of various inflammatory skin diseases, the therapeutic potential of fatty acids has been investigated. Among fatty acids, ω-3 and ω-6 fatty acids are recognized as essential fatty acids that cannot be synthesized in the human body. Therefore, these fatty acids can be taken in only by eating foods containing them. Linoleic acid is a typical example of ω-6 fatty acids; ω-3 fatty acids, such as eicosapentaenoic acid (EPA) and docosapentaenoic acid (DHA), are abundant in fish oil. ω-6 fatty acids are metabolized to arachidonic acid and subsequently converted into inflammatory lipid mediators, such as leukotriene B4 (LTB4) and prostaglandin E2 (PGE2). On the other hand, α-linolenic acid is an omega 3 fatty acid found mostly in plant foods, such as walnuts and vegetable oils, and is metabolized to EPA and DHA, which are known to exhibit anti-inflammatory effects. Epidemiological studies in the 1970s have shown that ω-3 fatty acids reduce the risk of psoriasis and other inflammatory diseases [52]. The last few decades of research have focused on the involvement of these lipid mediators in cutaneous inflammation.

Since LTB4 is produced in the lesional skin of psoriasis, it has been speculated that LTB4 may exacerbate psoriasis. Psoriasis-like dermatitis is attenuated in mice lacking the LTB4 receptor BLT1 [53]. BLT1 deficiency inhibits neutrophil migration in addition to the contribution of LTB4 to the migration of dendritic cells [31] and the production of inflammatory cytokines such as IFN-γ [54] from lymphocytes. Some clinical studies have been conducted to clarify the possibility that suppressing LTB4 may have a beneficial impact on psoriasis. LTB4 production is suppressed by fish oil supplementation in patients with psoriasis [55]. In addition, another ω-6 fatty acid metabolite, thromboxane A2 (TxA2), enhances IL-17 production and promotes imiquimod-induced psoriatic skin inflammation in mice [56].

EPA itself has a suppressive effect on inflammatory cytokine production. EPA suppresses the production of TNF-α and IL-1β, and inflammatory lipid mediators, such as PGE2 and LTB4 [57]. Patients with psoriasis exhibit a lower intake of ω-3 fatty acids compared with healthy controls [10], and the metabolites of ω-3 fatty acids, resolvin E1, resolvin D1, and Maresin 1, exert anti-inflammatory actions in imiquimod-induced psoriasis-like skin inflammation in mice [58,59,60]. Impaired skin inflammation was also observed in approximately 80% of patients with psoriasis using fish oil supplements [61].

Recent epidemiological studies have shown controversial results regarding the therapeutic potential of ω-3 fatty acid supplementation for atopic dermatitis [62,63]. However, a recent systematic review revealed that 9 of 13 observational studies and 5 of 7 randomized control trials identified a beneficial relationship between increased intake of ω-3 fatty acids or fish and allergic disease incidence, especially atopic dermatitis [64]. Mouse experiments have shown the anti-inflammatory effects of ω-3 fatty acids [65] and their metabolites on atopic dermatitis-like skin inflammation [66]. DHA/EPA administration decreased the degree of skin inflammation in an atopic dermatitis mouse model by decreasing the concentration of local LTB4 in the skin [65].

In contrast to those of ω-3 fatty acids, higher levels of prenatal ω-6 fatty acids were associated with an increased relative risk of atopic dermatitis in children (odds ratio: 1.25 (1.01–1.54)) [67]. However, there was no association between the risk of atopic dermatitis and prenatal ω3 fatty acids or ω6:ω3 fatty acids ratio. The skin of patients with atopic dermatitis contains high concentrations of LTB4 [68] (lesional atopic dermatitis: mean 5.2 ng/gm, healthy skin: mean <0.05 ng/gm). LTB4 is implicated in the recruitment of neutrophils and Th2 cells and is thought to play a pivotal role in the pathogenesis of acute inflammation in both human and murine models of atopic dermatitis [69]. In contrast, PGE2 also exists at high concentrations in the skin of patients with atopic dermatitis (lesional AD skin: mean 97.2 ng/gm, healthy skin: mean 27.1 ng/gm) [68] and PGE2-EP2 signaling enhances the protective effect against atopic dermatitis by suppressing TSLP production [70]. Accordingly, cyclooxygenase (COX)-2 inhibition enhances Th2 immune reactions triggered by atopic dermatitis-like skin inflammation [71].

Oral administration of DHA and EPA reduces skin swelling, inflammatory cytokine production, and inflammatory cell infiltration [72,73]. An EPA-derived metabolite, resolvin E1, regulates dendritic cell migration in the skin [31,74], and impairs subsequent Th1 immune response and tissue inflammation [31]. Previous studies on a mouse model have reported the importance of ω-6 fatty acids. The metabolite PGE2 contributes to the pathogenesis of contact hypersensitivity via specific receptors. PGE2-EP3 enhances contact hypersensitivity [75], and PGE2-EP4 signaling promotes skin inflammation by upregulating the migration and maturation of Langerhans cells [76]. PGE2-EP2/EP4 signaling enhances IL-22 production by T cells and promotes contact dermatitis [77].

### 3.4. Smoking

Smoking causes many physiological and pathological adverse effects on human health and increases mortality associated with health risks such as malignancies and cardiovascular events [78]. In addition to these risks, inflammatory skin diseases are closely related to smoking.

A cross-sectional study conducted in 1985 reported the relationship between smoking and the risk of psoriasis [79]. Further investigations were conducted to elucidate the actual impact of smoking on the pathogenesis of psoriasis. Current and past smokers have been identified as under risk for psoriasis, unlike non-smokers [80]. Compared with non-smokers, smoking more than 11 pack-years increased the risk of psoriasis and past smoker quiet less than 19 years ago was also significantly increased the risk; however, there was no significant difference in the risk between never smoker and past smoker more than 20 years ago [80]. Another approach evaluated the contribution of smoking duration and volume to the risk of psoriasis, and found that longer smoking periods and higher smoking volume increased the risk of developing psoriasis [81]. It is speculated that nicotine may be an exacerbating factor for psoriasis as it induces inflammatory cytokines and increases angiogenesis and the proliferation of keratinocytes [82]. It has also been reported that smoking itself increases the proportion of IL-17-producing cells in peripheral blood and other systemic organs [83], suggesting that smoking may increase the prevalence of IL-17-producing cells in the skin as well. Therefore, in addition to the nicotine-mediated exacerbation of inflammation, smoking exacerbates skin inflammation in psoriasis through the activation of Th17 cells.

Smoking is closely related to the risk of atopic dermatitis [84,85,86]. In addition, heated tobacco products are associated with the risk of atopic dermatitis [87]. *Staphylococcus aureus* is a representative microorganism that exacerbates atopic dermatitis skin inflammation [88], and smoking increases the risk of *S. aureus* infection in patients with atopic dermatitis [89]. Smoking exposure also increases the risk of atopic dermatitis in children [90,91]. Cigarette smoking enhances the production of the Th2 cytokine IL-13 [92], and cigarette smoke extract enhances Th2 polarization mediated by T-cell immunoglobulin mucin-3 (TIM3) in dendritic cells in an Extracellular Signal-regulated Kinases (ERK)-dependent manner [93].

Smoking increases the risk of contact dermatitis [94,95] and hand eczema [96,97]. The mechanism is still controversial in part [98]; however, cigarettes enhance the Th1 immune response and IFN-γ cytokine production [99,100,101], whereas nicotine itself seems to suppress the immune reaction, including dendritic cell maturation and T cell activation and migration [102].

### 3.5. Alcohol

As humans cannot store alcohol as energy in the body, almost all alcohol is rapidly metabolized by the liver. There are two major enzymes involved in the metabolism of alcohol in the body—namely, alcohol dehydrogenase and aldehyde dehydrogenase. Ethanol is converted to acetaldehyde and finally to acetic acid by aldehyde dehydrogenase. Therefore, when considering the health effects of alcohol, it is necessary to keep in mind the effects of both ethanol and its metabolite, acetaldehyde, on the human body. Alcohol is known to have various effects on inflammatory skin diseases.

Considering the association between psoriasis and alcohol intake, some epidemiological studies have reported alcohol as an exacerbating factor for psoriasis [103]. The alcohol component, ethanol, exacerbates the pathology of psoriasis by inducing keratinocyte proliferation through the activation of keratinocyte cyclin D1 and keratinocyte growth factor, which can lead to epidermal thickening in psoriasis [104]. Ethanol also stimulates the production of inflammatory cytokines by keratinocytes, leading to the exacerbation of psoriasis inflammation [105]. In addition, ethanol itself stimulates lymphocyte proliferation in patients with psoriasis, but doses do not affect healthy individuals [106]. Therefore, it is considered that ingestion of alcohol contributes to skin inflammation, leading to epidermal thickening, production of inflammatory cytokines from keratinocytes, and local proliferation of lymphocytes in the skin.

Several epidemiological studies have identified a statistical relationship between alcohol intake and atopic dermatitis. A recent meta-analysis revealed a positive correlation between alcohol intake during pregnancy and the development of atopic dermatitis in children [107]. However, there was no significant association between alcohol intake and atopic dermatitis in both adults and adolescents. Histamine is a trigger that causes skin itch [108] and enhances skin inflammation in atopic dermatitis caused by mechanical skin damage due to scratching [109]. Antipruritic agents are commonly used for the treatment of atopic dermatitis, and effectively inhibit scratching behavior and skin inflammation [110]. Concanavalin A (con A) enhances histamine release, which is suppressed by ethanol in the acute phase [111], but not in chronic ethanol administration [112]. However, the metabolite of ethanol, acetaldehyde, enhances histamine release by mast cells and bronchial contraction, which is canceled by the anti-histamine drug [113].

There is no clear evidence for the contribution of alcohol to the development of contact dermatitis; however, vascular permeability is upregulated by alcohol intake [114,115], and this contributes to the development of tissue inflammation in the skin, which is not limited to contact dermatitis. Therefore, the acute inflammatory response to contact dermatitis might be exacerbated by alcohol intake.

### 3.6. Sleep

The circadian rhythm is a physiological phenomenon that fluctuates in a cycle of every 24 h and is present in the most fundamental behaviors of animals and plants. The circadian rhythm is generally referred to as a biological clock, and recent studies have identified its association with various diseases, such as lifestyle-related illnesses. The human circadian rhythm has a 25 h cycle, which is different from the daily cycle of the Earth, and the adjusting function of the circadian rhythm is important for adaptation to the daily cycle of the Earth. Previous epidemiological studies have identified sleep disturbance as a possible risk factor for inflammatory skin diseases.

Epidemiological studies have revealed the relationship between shift work and psoriasis risk. A prospective study reported that shift workers had a higher risk of psoriasis compared with daytime workers [116]. There is a clock gene that controls circadian rhythm; however, imiquimod-induced psoriatic eruption is attenuated in mice lacking this clock gene, unlike in normal mice. Mice deficient in clock genes exhibit a lack of epidermal thickening, characteristic of psoriasis, upon histological examination, and IL-17 production is reduced as a result of a decreased IL-23 receptor expression [117]. Based on this result, it was expected that the clock gene would work normally by activating the circadian rhythm in day workers. On the other hand, the clock gene in shift workers is activated more than that in day workers due to circadian rhythm disturbances. Therefore, psoriatic skin inflammation may be exacerbated as the IL-23 receptor expressed in IL-17-producing cells is overexpressed in shift workers.

Poor sleep conditions are closely related to skin itch and dry skin [118]. Patients with atopic dermatitis who received melatonin supplementation showed a reduction in the severity of the atopic dermatitis score (SCORAD) compared with those who received a placebo [119]. Circadian clock genes regulate skin hydration, and stratum corneum hydration mediated by aquaporin-3 dysfunction is impaired in clock gene knockout mice [120].

A mouse experiment revealed the relationship between contact dermatitis and sleep; however, an epidemiological study provided no clear evidence. Ear swelling, serum immunoglobulin E levels, and mast cell numbers were enhanced in CLOCK mutant mice compared with those in wild-type mice. The serum corticosterone levels were lower in CLOCK mutant mice than in wild-type mice [121].

### 3.7. Obesity

Subcutaneous fat serves a survival function by acting as an energy storage medium. However, human beings do not necessarily need an energy storage function in the current environment, and the result of excess energy storage, obesity, enhances various risks to human health. Recent studies have shown that obesity is closely associated with inflammatory skin diseases. Dysfunction of lymphatic vessels reduces their clearance function and prolongs inflammation, resulting in exacerbation of skin inflammation in obese mice [122]. In actual clinical practice, cellulitis frequently occurs and becomes more severe in patients with lymphedema due to obesity [123].

Obesity is known to exacerbate inflammation. There is a statistically significant correlation between body mass index and psoriasis [124]. In addition, eruptions are exacerbated in psoriasis models on a high-fat diet, which enhances Th17 cell activation [125,126].

A previous statistical analysis showed the relationship between atopic dermatitis and obesity [127,128]. However, the underlying mechanism remains unclear. Obesity is responsible for changes in skin barrier function [129]. In addition, obese mice on a high-fat diet show an increase in TSLP production, which is an important driver of atopic dermatitis skin inflammation [70].

The statistical significance of the correlation between obesity and contact dermatitis remains unclear [95,130]. However, several recent reports have revealed a possible pathogenic role of obesity. Mice fed a high-fat diet showed impaired lymphatic vessel function due to leaky capillary lymphatics and decreased collecting vessel pumping capacity [131,132]. Consistently, obese mice showed increased skin inflammation in a contact dermatitis model and delayed clearance of inflammatory responses [122]. Vascular endothelial growth factor-C contributes to the development of skin inflammation in obese individuals, and intradermal injection of recombinant vascular endothelial growth factor-C enhances lymphangiogenesis and lymphatic function, leading to the suppression of cutaneous inflammation.

### 3.8. Others

Patients with intrinsic atopic dermatitis exhibit high serum concentrations of nickel [133], indicating that nickel intake might be involved in the development of atopic dermatitis skin lesions. In the Earth, nickel is abundantly present in soil (5–500 μg/g); the amounts in plant and animal tissues are 0.5–5 μg/g and 0.1–5 μg/g, respectively [134]. Nickel is present in high amounts in foods such as chocolate (27.87 mg/kg), crisps (12.70 mg/kg), and nuts (2.5 mg/kg) [134]. Since there are a limited number of reports on the beneficial impact of nickel-restricted food intake in the clinical setting, its clinical application cannot be recommended. Further investigation is required to clarify its beneficial effects on atopic dermatitis.

In addition, ultraviolet light exposure is one of the environmental factors [135] and is closely related to skin homeostasis [136]. Ultraviolet light exposure induces cytokines and corticotropin-releasing hormone, which exert systemic effects, including immunosuppression [135]. Actually, psoriasis and atopic dermatitis are commonly applied for the treatment of phototherapy, especially narrow-band UVB and psoralen and ultraviolet A (PUVA).

## 4. Summary of Inflammatory Reactions to Daily Lifestyle Factors

We have summarized the inflammatory cytokine and chemokine reactions to daily lifestyle factors in Table 1. Gluten or wheat protein, smoking, alcohol, sleep disturbance, and obesity can drive the Th1/Th2/Th17 immune response, whereas dietary fiber and its derivatives, short-chain fatty acids, and ω-3 fatty acids negatively regulate inflammatory reactions. Among ω-6 fatty acids, LTB4 and TxA2 positively regulate cytokine reactions; however, the effect of PGE2 depends on specific receptors. Since some parts of inflammatory cytokine reactions remain unclear, the role of these daily lifestyle factors in inflammatory skin diseases needs to be clarified.

## 5. Conclusions

This review summarizes daily lifestyle-related effects on representative inflammatory skin diseases. Since daily lifestyles affect human health through accumulation, knowledge of related skin inflammation may contribute to future treatment through lifestyle-related guidance that does not rely solely on drugs, in addition to preventing the onset of diseases. When using the knowledge gained from this review, it is very important to ask questions about daily lifestyles concerning what is needed to improve lifestyle problems. Further investigations are required to elucidate the detailed molecular mechanisms of daily lifestyle-related inflammatory skin diseases and develop some convenient tools to identify problems associated with daily lifestyles in patients with inflammatory skin diseases.

## Figures and Tables

**Table 1:** Summary of inflammatory reaction in each skin inflammation disease model.

	Psoriasis	AD	Contact Dermatitis
Gluten or wheat	Gluten free diet CD4^+^ cell infiltration ↓ [34] IFN-γ, IL-12, IL-17 ↓ [35]	Gluten: TSLP ↑ [40] Salt soluble wheat protein: TNF-α, IFN-γ, IL-1β, IL-4, IL-10, IL-13, IL-17, CCL1, CCL5, CCL11, CCL12, CCL22, CCL24, CXCL9, CXCL16, E-selectin ↑ [39]	
Fiber	Dietary fiber: Treg ↓ [45] Butyrate:IL-17, IL-6↓IL-10 ↑ [46]		Butyrate: Treg ↑ IL-6 ↓ [49], IL-10 ↑ [49,50].
Lipid	LTB4: Neutrophil migration, IL-19 ↑ [53], DC migration↑ [31], IFN-γ ↑ [54] TxA2: IL-17 ↑ [56]. EPA: TNF-α, IL-1β ↓ [57]. RvE1: IL-17, IL-23, IL-12b ↓ [58] MaR1: IL-17 ↓ [59] RvD1: IL-23, IL-22, IL-17, TNF-α ↓ [60].	EPA/DHA: IL-13, IL-17, LTB4, T cell migration ↓ [65] RvE1: IFN-γ, IL-4, IgE ↓ [66] LTB4: IL-4, IL-13, CD4 migration↑ [69] PGE2-EP2: TSLP ↓ [70]	DHA: IFN-γ, IL-6, IL-1β, IL-2↓ [72] EPA/DHA: TNF-α, IL-2, IL-4, IL-12 ↓ IL-10 ↑ [73] RvE1: DC migration ↓ [31,74], IFN-γ ↓ [31] PGE2-EP3: CXCL1 ↓ [75] PGE2-EP4: LC maturation, migration ↑ [76]. PGE2-EP2/EP4: IL-22 ↑ [77]
Smoking	IFN-γ, IL-17A ↑ [83]	IL-13 ↑ [92],, IL-4 ↑ [93]	IFN-γ ↑ [99,100,101] Nicotine: DC maturation, T cell activation/migration ↓ [102]
Alcohol	Ethanol: IFN-γ, TNF-α, IL-6 ↑ [105], Lymphocyte proliferation ↑ [106]	Ethanol: Histamine ↓ [111] Acetaldehyde: Histamine ↑ [113]	
Sleep disturbance	IL-17 ↑ [117]	Skin hydration ↓ [120]	Mast cell, IgE↑ [121]
Obesity	Th17 ↑ [125,126]	Skin burrier ↓ [129], TSLP ↑ [70]	Lymphatic vessel disfunction [131,132], IFN-γ ↑ [122]

Note: ↑: increase, ↓: decrease.

## Data Availability

Not applicable.

## Abbreviations

CCL	C-C motif chemokine ligand
CXCL	C-X-C motif ligand
DC	dendritic cell
EPA	eicosapentaenoic acid
DHA	docosahexaenoic acid
IFN	interferon
IL	interleukine
LTB4	leukotriene B4
MaR1	maresin 1
PGE2	prostaglaind E2
RvD1	resolvin D1
RvE1	resolvin E1
TNF	tumor necrosis factor
TSLP	Thymic stromal lymphopoietin
TxA2	thromoboxane A2
